# A cholera outbreak in a rural north central Nigerian community: an unmatched case-control study

**DOI:** 10.1186/s12889-018-6299-3

**Published:** 2019-01-25

**Authors:** Chioma Cindy Dan-Nwafor, Uzoma Ogbonna, Pamela Onyiah, Saheed Gidado, Bashorun Adebobola, Patrick Nguku, Peter Nsubuga

**Affiliations:** 1 Nigeria Field Epidemiology and Laboratory Training Programme, Abuja, Nigeria; 2 African Field Epidemiology Network, Kampala, Uganda; 3 Global Public Health Solutions, Atlanta, USA

**Keywords:** Unmatched case control study, Cholera outbreak, Hand hygiene, Nigeria

## Abstract

**Background:**

Cholera remains a disease of public health importance in Nigeria associated with high morbidity and mortality. In November 2014, the Nigeria Field Epidemiology and Laboratory Training Programme (NFELTP) was notified of an increase in suspected cholera cases in Gomani, Kwali Local Government Area. NFELTP residents were deployed to investigate the outbreak with the objectives of verifying the diagnosis, identifying risk factors and instituting appropriate control measures to control the outbreak.

**Methods:**

We conducted an unmatched case-control study. We defined a cholera case as any person aged ≥5 years with acute watery diarrhea in Gomani community. We identified community controls. A total of 43 cases and 68 controls were recruited. Structured questionnaires were administered to both cases and controls. Four stool samples from case-patients and two water samples from the community water source were collected for laboratory investigation. We performed univariate and bivariate analysis using Epi-Info version 7.1.3.10.

**Results:**

The mean age of cases and controls was 20.3 years and 25.4 respectively (*p* value 0.09). Females constituted 58.1% (cases) and 51.5%(controls). The attack rate was 4.3% with a case fatality rate of 13%. Four stool (100%) specimen tested positive for *Vibrio cholerae*. The water source and environment were polluted by indiscriminate defecation. Compared to controls, cases were more likely to have drank from Zamani river (OR 14.2, 95% CI: 5.5–36.8) and living in households(HH) with more than 5 persons/HH (OR 5.9, 95% CI: 1.3–27.2). Good hand hygiene was found to be protective (OR 0.3, 95% CI: 0.1–0.7).

**Conclusion:**

*Vibrio cholerae* was the cause of the outbreak in Gomani. Drinking water from Zamani river, living in overcrowded HH and poor hand hygiene were significantly associated with the outbreak. We initiated hand hygiene and water treatment to control the outbreak.

**Electronic supplementary material:**

The online version of this article (10.1186/s12889-018-6299-3) contains supplementary material, which is available to authorized users.

## Background

Cholera remains a global threat to public health and a key indicator of lack of social development. Cholera, an acute diarrheal disease caused by gram-negative bacillus *Vibrio cholerae of* serogroup 01 and 0139 is associated with high morbidity and mortality [1–3].

The onset of cholera often starts with stomach cramps, vomiting and diarrhoea, and if left untreated may progress to fluid losses of up to 1 litre per hour, resulting in severe dehydration and metabolic acidosis, and consequently kidney failure, shock, coma, and death. About 50% of cholera cases are asymptomatic. Asymptomatic cases shed vibrios in their stools and serve as a potential source of infection to others. Symptomatic patients may also shed vibrios before the onset of illness and will continue to shed the organisms for about 1 to 2 weeks [1, 3, 4].

Cholera is transmitted through the fecal-oral route via contaminated food, carriers and unsanitary environmental conditions. Cholera outbreaks tend to occur as a result of contamination of food or water with *Vibrio cholera* organisms due to poor personal hygiene, unsafe environmental sanitation conditions compounded by lack of potable water supply. Internal displacement of persons by natural and man-made disasters leading to unstable living conditions with contamination of food and water sources have also been reported to cause cholera outbreaks [5–7].

Globally an estimated 3 to 5 million cholera cases and 28,000 to 150,000 deaths occur yearly.

However, the infection is common to developing countries in the tropics and subtropics with high human poverty index [2, 8, 9]. Cholera is endemic in Africa, parts of Asia, Middle East, and South and Central America [10]. In Africa, there have been recurrent cholera outbreaks, characterized by a large disease burden and high case-fatality rates. African countries accounted for 3,316,201 (46%) of the suspected cholera cases reported to the WHO from 1970 to 2012. In 2012, sub-Sahara Africa recorded 71% of all reported cases and 86% of cholera deaths [11, 12]. In 2013, a total of 129,064 cases were notified from 47 countries, including 2102 deaths; the World Health Organisation (WHO) however believes the officially reported cases account for not more than 5–10% of the actual disease burden. The discrepancy between the reported figures and the estimated burden of the disease could be ascribed to poor surveillance and laboratory systems. Political motives such as fear of trade and travel sanctions have also been implicated [8, 9].

Nigeria is reported to be one of the three major current cholera foci in the world [13]. The first series of cholera outbreaks in Nigeria were reported between 1970 to 1990, subsequently recurrent outbreaks followed [14]. In 2010, Nigeria reported a total of 41,787 cases with 1716 deaths from 18 northern states with case fatality rate [CFR] of 4.1%. This CFR exceeded the mean overall CFR of 2.4% reported in Africa from 2000 to 2005 and the 1% WHO acceptable rate [2, 15]. The 2010 outbreak was attributed mainly to contamination of water supplies with diarrhoea discharge of untreated cholera patients during the rainy season.This therefore, brought to the focus the vulnerability of Nigerian rural communities [1].

On November 8, 2014, the head of the Primary Health Care Department(PHCD) through the Disease Surveillance and Notification Officer (DSNO) reported an increase in the number of reported cases of vomiting and diarrhea in Gomani village, Kwali Area Council, Federal Capital Territory (FCT) Abuja. The Nigeria Field Epidemiology and Laboratory Training Programme (NFELTP) was notified of the outbreak. An outbreak response team was immediately mobilized and deployed to Gomani settlement. The team investigated the outbreak with the objectives of verifying the diagnosis, identifying risk factors and instituting appropriate control measures to control the outbreak.

## Materials and methods

We conducted an unmatched case control study to identify potential risk factors of the outbreak. We conducted a laboratory analysis of stool and water samples from the community and instituted appropriate control measures.

### Study area

The case control study was conducted in Gomani settlement, Kundu ward of Kwali Local Government Area (LGA) Federal Capital Territory (FCT). Gomani has an estimated population of about 1000 people. The main economic activities amongst Gomani residents are farming, petty trading, and fishing. The Gurara and Zamani rivers serve as the major sources of drinking for most Gomani residents. Gomani settlement has one primary health center (PHC).

### Study population

Study participants constituted of 48 recently identified cholera cases and 68 community controls identified in in Gomani settlement Kwali area council, FCT. Cases and controls were recruited into the study using the following definitions.

#### Case definition

We defined a suspected case of cholera as “any person or patient aged 5 years and above with acute watery diarrhea with or without vomiting living in Gomani settlement from October 26, to November 9, 2014”.

#### Control definition

We defined a control as “any person living in Gomani aged 5 years and above without history of diarrhea October 26, to November 9, 2014”.

### Identification of cases and recruitment of controls

We obtained a line list of all cases (previously and currently) admitted at Gomani PHC from the Disease Surveillance and Notification Officer. A community active case search was conducted, and all cases meeting case definition were recruited. Information on age, sex, residence, date of onset of illness, signs, symptoms and outcome were obtained from cases and used to generate hypotheses about potential exposures that were common to the cases. In households with multiple cases, all cases were recruited in the study. Controls were systematically recruited in the community. Starting from households that reported cases, we visited the second household to the right of the case household. In the selected households all members of the household were listed, and 1 member selected randomly as a control. Only 1 control was selected even for case households with more than 1 case. Interviews excluded household members who had reported a history of vomiting and diarrhea.

### Sample size calculation

Using a 95% confidence interval, power of 80%, odds ratio (OR) of 4 and case to control ratio of 1:1.5, a sample size of 44 cases and 65 controls was calculated using Epi Info version 7.1.3.10 StatCalc. However, 43 cases and 68 controls met the inclusion criteria and were recruited for the study.

### Study instrument

Interviewers administered a structured questionnaire to cases and controls in English and Gbaygi languages. The questionnaire captured socio-demographic information, clinical information (for cases), risk factors, and knowledge, attitude, and practice on cholera.

### Laboratory investigations

We collected stool samples from four cases, and we tested the samples using cholera rapid diagnostic test kits.

### Environmental assessment

We collected water samples from Zamani and Gurara River. The samples were sent to National Hospital Laboratory Abuja for isolation of *Vibrio cholerae* using Thiosulfate Citrate Bile Salts Sucrose agar (TCBS) culture media.

We inspected Gomani community source of water supply which is principally Gurara and Zamani rivers. Activities and practices along these water bodies were noted. Drinking water storage facilities and waste management in the homes were also inspected.

## Data management

We conducted univariate and bivariate analysis using Epi Info 7.1.3.10. We characterized the data in person, place and time. We calculated cholera incidence by age and sex. The outbreak timeline was summarized as an epidemic curve. Cases were compared with controls by calculating of odds ratio with 95% confidence intervals. We computed the attack rate using the formula (AR: cases/100,000 population) and the case fatality rate (CFR: deaths /cases).

## Ethical considerations

Informed oral consent were obtained from the participants before the interviews because the outbreak was in rural setting and most respondents were uneducated and unable to read and write. Confidentiality of the respondents were ensured through data coding. Due to the exigencies of the response, ethics approval was waived by Federal Capital Territory Health Research Ethics Committee. Permission was obtained from the department of public health Kwali Local Government Area (LGA) during the response and preliminary report of the outbreak was discussed with Gomani community leader and Kwali LGA public health team.

## Results

A total of 111 participants were recruited for the case-control study of which 43 were cases and 68 controls. The mean age of cases and controls was 20.3 years and 25.4 respectively (*p* value 0.09). Females constituted 58.1% (cases) and 51.5% (controls). The proportion of those aged 30 years and above was 20.9% among cases and 35.3% among the controls. Overall 9% of the study participants had secondary education (cases 7%, controls 10.3%). Nearly two thirds of the study participants were of Muslims faith (cases 60.5%, controls 72.1%). Farming was the most predominant occupation with 60.5% of cases being farmers compared to 72.1% of controls. (Table 1).

**Table 1:** Socio-demographic distribution of cases and controls in Gomani, November 2014

Characteristics	Cases *n* (%)	Controls *n* (%)	Total *n* (%)
Total	43 (38.7)	68 (61.3)	111(100)
Sex			
Female	25 (58.1)	35 (51.5)	60 (54.1)
Male	18 (41.9)	33 (48.5)	51 (45.9)
Age Group			
5–9	16 (37.2)	11 (16.2)	27(24.3)
10–19	3 (7.0)	14 (20.6)	17(15.3)
20–29	15 (34.9)	19 (27.9)	34 (30.6)
30 & above	9 (20.9)	24 (35.3)	33 (29.7)
Religion			
Christian	7 (16.3)	28 (41.2)	35 (31.5)
Muslim	32 (74.4)	38 (55.9)	70 (63.1)
Traditionalist	4 (9.3)	2 (2.9)	6 (5.4)
Occupation			
Petty trader	0 (0.0)	3 (4.4)	3(2.7)
Farmer	26 (60.5)	49 (72.1)	75(67.6)
House wife	0 (0.0)	2 (2.9)	2 (1.8)
Student	17(39.5)	14 (20.6)	31 (27.9)
Education			
None	22 (51.6)	42 (61.8)	64 (57.7)
Primary	18 (41.9)	19 (27.9)	37 (33.3)
Secondary	3 (7.0)	6 (8.8)	9 (8.1)
Islamic	0 (0.0)	1 (1.5)	1 (0.9)

**Fig. 1:**
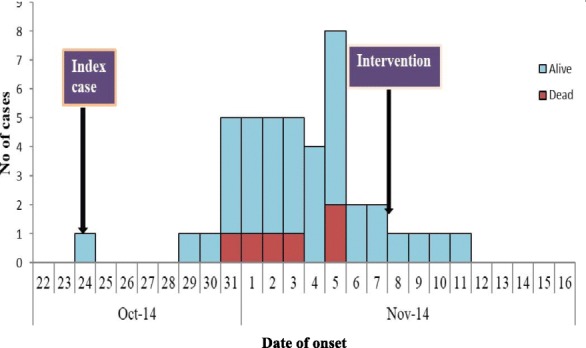
Epidemic curve of Cholera cases in Gomani, October to November, 2014

Figure 1 shows the epidemic curve of the outbreak. The index case was reported on the 24th of October with subsequent cases being reported within 5 days of the index case reporting symptoms. The outbreak peaked on the 5th of November and the last case was reported on 11th November. The 6 cases that succumbed to death were reported in the second week of the outbreak and occurred before commencement of the response to the outbreak. The response was initiated on 6th of the November 2014 when the national authorities were notified of the outbreak. The outbreak was controlled on 12th November.

**Fig. 2:**
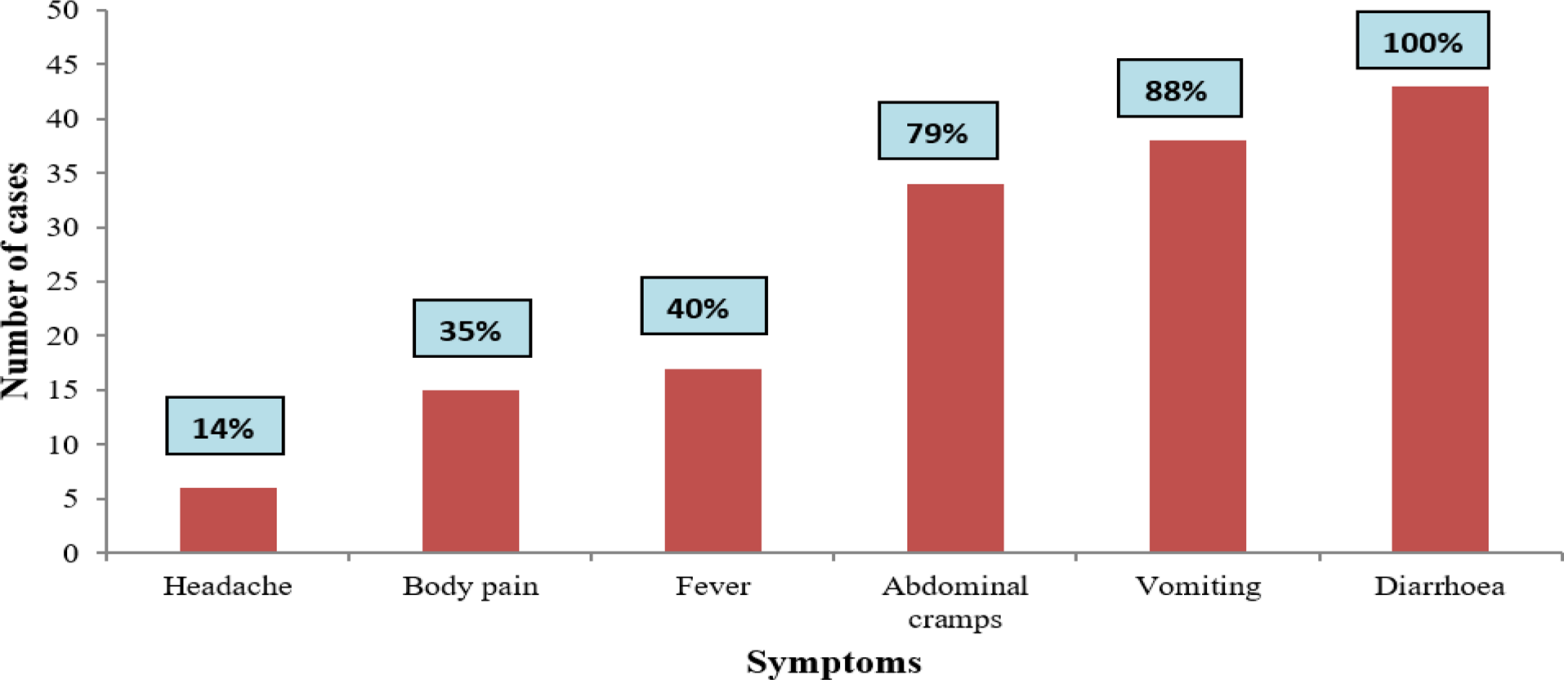
Signs and symptoms of Cholera cases in Gomani, November, 2014

The most frequently reported symptoms by the cases was diarrhea 43(100%), vomiting 38 (88%), abdominal cramps 34 (79%) and fever 17(40%). The least symptom experienced by the cases was headache 6 (14%) (Fig. 2). The overall cholera attack rate was 4.3% with 43 cases and 6 deaths, case fatality rate of 14% among 1000 residents of Gomani settlement.

Compared to the controls (81.4%), cases (23.5%) were more likely to have Zamani river as their drinking water source than Gurara river/others sources (OR 14.2, 95% CI: 5.5–36.8). Washing hands with soap or ash before eating was found to be a protective factor as controls had a 70% reduction in risk of acquiring the disease following hand washing (OR 0.3, 95% CI:1.4–10.3). Compared to controls, cases were 6 times more likely to come from households with > 5 persons (OR 5.9, 95% CI: 1.3–27.2) (Table 2).

**Table 2:** Risk factors of Cholera outbreak in Gomani, November 2014

Risk Factors	Cases (43) *n* (%)	Controls (68) *n* (%)	OR	95% CI
Drinking water source				
River Zamani	35 (81.4)	16 (23.5)	14.2	5.5–36.8
River Gurara	8 (18.6)	52 (76.5)	-	-
Hand washing with soap/ash before eating	6 (14.0)	26 (38.2)	0.3	0.1–0.7
> 5 persons per household	40 (93.0)	51 (75.0)	5.9	1.3–27.2
Drinking water storage container without cover	2 (4.7)	1 (1.5)	3.2	0.3–36.1
No formal education	22 (51.2)	42 (61.8)	0.6	0.3–1.4
Lack of rack for drying plates	11 (25.6)	15 (22.1)	1.2	0.5–2.9

All the four stool specimens tested positive for *V.cholera* using rapid test kit while Gurara and Zamani river samples yielded material growth of *coliforms*. Gurara River was found to be macroscopically clean with high current flow, unlike the stagnant Zamani river polluted due to, mass bathing, washing of clothes and indiscriminate defecation in and around the river bank. The community had only three non-functional boreholes at the time of the study. We observed that majority (97%) of the residents defecate at the bushes behind their houses, and 3% used shallow pit latrines. Waste management was found to be poor with refuge heaps littered around houses.

## Discussion

Epidemiologic and environmental evidence indicated that the cholera outbreak resulted from drinking water from Zamani River. Poor personal hygiene and overcrowding were also identified as major risk factors for acquiring the disease. These findings agree with similar studies in Nigeria [2, 10]. However, many cholera outbreaks in Nigeria are not epidemiologically investigated to identify risk factors for the illness [16, 17] and therefore control measures are empiric without addressing specific risk factors associated with the outbreak. Lack of potable drinking water and insufficient awareness/education of communities on practical drinking water treatment strategies has kept many of the rural communities at risk of cholera once introduced in their communities.

The cholera epidemic affected all age groups in the community; although, age group 5–9 years had the highest proportion of cases. Our findings are consistent with the study done in Nigeria but contrary to the study done in Nepal where ≤5-year-olds were mostly affected although our study excluded this age group [2, 18].

The epidemic curve suggested a common source; household contact could be an underlying factor for community-wide transmission of this outbreak. It’s possible the main source of drinking water in the community was contaminated and many of the community members exposed to infection. Given than only 5–10% of cholera cases present with classical symptoms it’s likely that nearly half of the community members were infected. Deaths occurred earlier in the outbreak before response activities were instituted. Response activities would have been more effective in preventing cases and death if it was instituted timely. However, timely response is dependent on timely notification and confirmation of the outbreak hence the need for a more sensitive community-based reporting of public health events.

Washing of hands with soap and water before eating a meal was found to be a protective factor in our study. Similar findings by other studies have also indicated that use of soap and hand washing promotion can achieve a 26 to 62% decrease in the incidence of diarrhoea in developing countries [17, 19, 20]. Likewise, the 1995–1996 cholera outbreaks in Kano state were also attributed to not washing hands with soap before eating food [21]. This indicates that risk communication gaps still exists.

Cholera has been termed the “disease of poverty” since social risk factors play an important role in its transmission [22, 23]. Significant association of cholera infection with overcrowding in our study is concomitant with this fact; moreover, our environmental assessment revealed poor environmental sanitation infrastructure like indiscriminate defecation in the environment due to lack of toilet facilities and improper waste management, these are conditions highly correlated with poverty and low socio-economic status.

Cholera exists as a seasonal disease in most countries [24]. In Nigeria, cholera infections have been reported in both rainy and dry seasons, although the burden of cholera tends to increase during beginning of rainy and dry seasons [1, 25]. The Gomani cholera outbreak occurred during the dry season, similar to the pattern observed in Calabar, South-southern part of Nigeria, where cholera outbreaks mostly occurred during the dry season [26].This could be attributed to scarcity of potable water during the dry season and therefore the tendency of people to obtain drinking and cooking water from alternative sources with higher risk of contamination which includes stagnant water bodies.Our study highlighted these findings in that cases were likely to obtain drinking water from stagnant Zamani river due to its proximity as opposed to Gurara river [1].

Control of cholera outbreaks requires effective surveillance and response systems which are often sub-optimal in developing countries and therefore this study accentuates the need for an effective surveillance system with the capacity to appropriately detect and contain cholera outbreaks timely [2, 27].

The long-term solution for cholera control lies in economic development through universal access to safe drinking water and adequate sanitation [28, 29]. Crucial cholera epidemic preventive mechanism remains providing a waste management system that separates waste from the water supply [30]. Oral cholera vaccines (OCV) which are additional efficient tool to control cholera outbreaks are yet uncommonly used in Nigeria [8]. OCVs though not a replacement for conventional control measures like portal safe water and personal hygiene, could serve as a complementary measure [31, 32].

In Guinea, two complete doses of cholera vaccine during an outbreak was found to be associated with significant protection against cholera with 86.6% vaccine effectiveness [33]. Vaccine safety and conferment of 85% immunity for 4–6 months in all age groups was found in Bangladesh and Peru field cholera vaccine trials done, similarly another field trial in Kolkata, India obtained 65% vaccine efficacy of up to 5 years [8].

This finding serves as supporting evidence on the addition of vaccination as part of the response to cholera outbreaks and the need to plan and implement regular cholera vaccination programmes in cholera endemic countries such as Nigeria [33, 34].

## Limitations

This study was also burdened with the several limitations including late notification of the outbreak which could be attributed to remote, poor access road network to Gomani settlement evidently delayed initiation of response. Nevertheless, response was commenced by the 6th of the November 2014 though not timely but mitigated the outbreak.

Health care workers strike action delayed the laboratory culture investigation and contributed to the failure to isolate *V. cholera* from the water samples. Lastly, we could not entirely rule out the possibility of misclassification of cases as controls since most cholera cases are asymptomatic. However, we tried to minimize this selection bias by recruiting our controls from every two households to the right of the household of the cases where no member had no signs and symptoms of diarrheal disease within the study period. Only recent cases were recruited for the study. Furthermore, confounders such as socioeconomic status and differences in age groups in the unmatched case-control study could have influenced the association found.

Despite these limitations, the study provided useful information to stakeholders on actions that will avert future outbreaks by provision of basic water, sanitation and hygiene infrastructures such as functional boreholes and standard pit latrines. Community risk communication and surveillance strategies need significant improvements to ensure prevention of adverse effects of diarrheal diseases in general and cholera in specific.

## Conclusion

An outbreak of cholera was investigated in Gomani Settlement Kwali Local Government Area of FCT. We established that drinking water from Zamani river was the major source of the outbreak. Poor personal hygiene and overcrowding were also identified as risk factors.

On the interim health education on proper hand hygiene and chlorination of water were initiated based on our recommendation and this controlled the outbreak.

Implementation of targeted interventions such as rehabilitation of existing boreholes, construction of standard pit latrines and the establishment of proper waste disposal systems are long-term sustainability measures to prevent future outbreaks.

## Additional files

Additional file 1: Cholera Gomani outbreak investigation questionnaire data (111) translated to xls.doc. (XLS 173 kb)

Additional file 2: Cholera Gomani outbreak investigation case control questionnaire sample. (DOCX 93 kb)

## Abbreviations

AR: Attack rate
CFR: Case fatality rate
CI: Confidence interval
DSNO: Disease Surveillance and Notification Officer
FCT: Federal Capital Territory
HH: Household
LGA: Local Government Area
NFELTP: Nigeria Field Epidemiology and Laboratory Training Programme
OCV: Oral cholera vaccines
OR: Odds ratio
PHC: Primary health center
SD: Standard deviation
TCBS: Thiosulfate Citrate Bile Salts Sucrose
WHO: World Health Organisation

## References

[1] Adagbada OA, Adesida AA, Nwaokorie OF, Niemogha M-T, Coker AO. Cholera epidemiology in Nigeria an overview. Pan Afr Med J. 2012;59:1–12. PMC3428179 22937199

[2] Dalhat MM, Isa NA, Nguku P, Nasir SG, Urban K, Abdulaziz M, Dankoli RS, Nsubuga P, Poggensee G (2014). Descriptive characterization of the 2010 cholera outbreak in Nigeria. BMC Public Health.

[3] Yamai S, Okitsu T, Shimada T, Katsube Y (1994). Distribution of serogroups of Vibrio cholerae non-O1 non-O139 with specific reference to their ability to produce cholera toxin and addition of novel serogroups. J Japan Assoc Infect Dis.

[4] Kaper JB, Morris JG, Levine MM (1995). Cholera. Clin Microbiol Rev.

[5] Qadri F (2005). Enterotoxigenic *Escherichia coli* and *Vibrio cholerae* diarrhea, Bangladesh, 2004. Emerg Infect Dis.

[6] Shikanga OT, Mutonga D, Abade M, Amwayi S, Ope M, Limo H, Mintz ED, Quick RE, Breiman RF, Feikin DR (2009). High mortality in a cholera outbreak in western Kenya after post-election violence in 2008. Am J Trop Med Hyg.

[7] Siddique AK, Islam Q, Akram K, Mazumder Y, Mitra A, Eusof A (1989). Cholera epidemic and natural disasters; where is the link. Trop Geogr Med.

[8] Ali M, Lopez AL, You YA, Kim YE, Sah B, Maskery B (2012). The global burden of cholera. Bull World Health Organ.

[9] Saulat Jahan (April 13th 2016). Cholera – Epidemiology, Prevention and Control.IntechOpen. 2016. 10.5772/63358.

[10] Akyala IA, Bright ES, Olufemi A, Adebola O, Nguku P (2014). Investigation of cholera outbreak in an urban north central Nigerian community-the Akwanga experience. Public Health Res.

[11] Mengel MA (2014). Cholera in Africa: new momentum in fighting an old problem. Trans R Soc Trop Med Hyg.

[12] Mengel MA, Delrieu I, Heyerdahl L, Gessner BD (2014). Cholera outbreaks in Africa. Curr Top Microbiol Immunol.

[13] Piarroux R, Faucher B (2012). Cholera epidemics in 2010: respective roles of environment, strain changes, and human-driven dissemination. Clin Microbiol Infect.

[14] Lawoyin TO, Ogunbodede NA, Olumide EAA, Onadeko MO (1999). Outbreak of cholera in Ibadan, Nigeria. Eur J Epidemiol.

[15] World Health Organization. Global task force on cholera control. In: Weekly epidemiological Record.Cholera articles: WHO. 2010;85(31):293–308.

[16] Tauxe RV, Mintz ED, Quick RE (1995). Epidemic cholera in the new world: translating field epidemiology into new prevention strategies. Emerg Infect Dis.

[17] Hutin Y, Luby S, Paquet C (2003). A large cholera outbreak in Kano City, Nigeria: the importance of hand washing with soap and the danger of street-vended water. J Water Health.

[18] Ise T, Pokharel BM, Rawal S, Shrestha RS, Dhakhwa JR (1996). Outbreaks of cholera in Kathmandu Valley in Nepal. J Trop Pediatr.

[19] Shahid NS, Greenough WB, Samadi AR, Huq MI, Rahman N (1996). Hand washing with soap reduces diarrhoeaand spread of bacterial pathogens in a Bangladesh village. J Diarrhoeal Dis Res.

[20] Pinfold JV, Horan NJ (1996). Measuring the effect of a hygienebehaviour intervention by indicators of behaviour and diarrhoeal disease. Trans R Soc Trop Med Hyg.

[21] Lipp EK, Huq A, Colwell RR (2002). Effects of global climate on infectious disease: the cholera model. Clin Microbiol Rev.

[22] Charles RC, Ryan ET (2011). Cholera in the 21^st^ century. Curr Opin Infect Dis.

[23] Snowden FM (2008). Emerging and reemerging diseases: a historical perspective. Immunol Rev.

[24] Rebaudet S, Sudre B, Faucher B, Piarrou R (2013). Cholera in coastal Africa: a systematic review of its heterogeneous environmental determinants. J Infect Dis.

[25] Umoh JU, Adesiyun AA, Adekeye JO (1983). Epidemiological features of an outbreak of gastroenteritis/cholera in Katsina, Northern Nigeria. J Hyg Camb.

[26] Ndon JA, Udo SM, William B (1992). *Vibrio*-associated gastroenteritis in the lower Cross-River basin of Nigeria. J Clin Microbiol.

[27] Vugia DJ, Koehler JE, Ries AA (1992). Surveillance for epidemic cholera in the Americas: an assessment. MMWR CDC Surveill Summ.

[28] Nygren BL, Blackstock AJ, Mintz ED (2014). Cholera at the crossroads: the association between endemic cholera and National Access to improved water sources and sanitation. Am J Trop Med Hyg.

[29] Date K, Person B, Nygren B, Were V, Kola S, Ayers T, Quick R (2013). Evaluation of a rapid cholera response activity—Nyanza Province, Kenya 2008. JID.

[30] Drazen JM, Klempner MS (2005). Disaster, water, cholera, vaccines, and hope. N Engl J Med.

[31] World Health Organization/Food and Agriculture Organization (2005). Risk assessment of choleragenic *Vibrio cholerae* 01 and 0139 in warm-water shrimp in international trade.

[32] WHO (2011). Prevention and control of cholera outbreaks:World Health Organisation policy and reccomendations.

[33] Luquero FJ, Grout L, Ciglenecki I, Sakoba K, Traore B, Heile M, Diallo AA, Itama C, Page A, Quilici M, Mengel MA, Eiros J, Serafini M, Legros D, Grais RF (2014). Use of *Vibrio cholerae* vaccine in an outbreak in Guinea. N Engl J Med.

[34] Chao DL, Hallorana ME, and Longini IM, Jr. Vaccination strategies for epidemic cholera in Haiti with implications for the developing world. Proc Natl Acad Sci USA. 2011;108:7081–5. 10.1073/pnas.1102149108 PMC3084143 21482756

