# Supplementary material for: A cholera outbreak in a rural north central Nigerian community: an unmatched case-control study
Source: BMC Public Health. 2019 Jan 25;19:112. doi: 10.1186/s12889-018-6299-3 (PMC6347749; doi:10.1186/s12889-018-6299-3)
Supplement: Supplementary file 2 — Cholera Gomani outbreak investigation case control questionnaire sample. (DOCX 93 kb) [file 12889_2018_6299_MOESM2_ESM.docx]

**Questionnaire**

**Cholera outbreak investigation Gomani FCT, November 2014**

Dear respondent

Good day, we are residents of the Nigerian Field Epidemiology and Laboratory Training Programme (NFELTP), Federal Ministry of Health, Abuja. We seek your permission to administer this questionnaire which is designed to assist us to investigate the current outbreak of cholera in your community and prevent future occurrences.

Please try to answer each question as sincerely as you can. This will help us achieve this objective. The information you provide will be treated with utmost confidence.

I hereby seek your consent to participate in the study.

Do you agree to participate in the study? Yes No

Thank you for your cooperation

**Section A: Socio - demographic details**

Questionnaire number _________ Interviewer’s name ___________

Interview date (dd/mm/yyyy) _______________________

1. Name__________________________________________________
2. Age (in years) ____________________________
3. Sex: Male Female
4. Occupation__________________________________
5. Educational Level: Nil Primary Secondary Tertiary

Others (specify) ___________________________

1. Religion: Christianity Islam African tradition

Others (specify) ________________________________

1. Settlement/village/household name ____________________________________
2. No of people in the household____________
3. Are you ill(diarrhoea)? Yes No If No, skip to question 16
4. If Yes, date of onset (dd/mm/yyyy) ________________

**Section B: Clinical information**

1. Symptoms of illness (select all that apply)
2. Diarrhoea
3. Vomiting
4. Abdominal cramps
5. Cramps in arms and legs
6. Fever
7. Headache

Others (specify) _____________________________________________

1. Duration of illness before seeking medical attention _______________
2. Treatment given at home __________________________
3. Treatment given at health unit

ORS IV fluids Tetracycline Co-trimoxazole Metronidazole

Others (specify) ___________________

1. Admitted for treatment at health facility? Yes No

Outcome: Alive Deceased

**Section C: Risk factors for Cholera**

1. Where does your household get drinking water?
2. Stream Gurara
3. River Zamani
4. Well
5. Pond
6. Borehole
7. Sachet water
8. Others (specify) _____________________
9. Does household prepare water before drinking? Yes No
10. If yes, how does household prepare water for drinking?

Boiling Chlorination

1. How does household store drinking water?
2. Drinking water stored in a container with cover
3. Drinking water stored in a container without cover
4. Please make a list of food items, including fruits taken in the last five days prior to this illness
5. Kosai
6. Pap/Kunu/Koko
7. Cold fried fish
8. Vegetable/Efo riri
9. Water melon
10. Moi moi
11. Beanssoup/gbegiri
12. Ayoyo
13. Tuwon dawa
14. Beans
15. Sugar cane
16. Orange
17. Banana
18. Miyan Kuka
19. Kubewa
20. Tuwon shinkafa
21. Tuwon masara
22. Balango
23. Suya
24. Miyan taushe
25. Indomie and egg
26. Doya
27. Others (specify) ________________________________
28. Do you always wash your hands with soap & water before eating? Yes No
29. Do you always wash your hands with soap & water after use of toilet? Yes No
30. Does the household have toilet facility? Yes No
31. If yes, what type?
32. Pit-latrine with cover
33. Pit-latrine without cover
34. Others (specify) ______________________________________
35. If no, where do you defecate?
36. Back yard
37. farm
38. bush
39. stream
40. Community Latrine
41. Ohers (specify)____________
42. How does your household dispose refuse?
43. Burning
44. Throwing to the backyard
45. Throwing into the bush
46. Throwing into stream
47. Others (specify) ___________________________________
48. Did you travel outside Gomani within one week prior to onset of your illness? Yes No
49. If yes, specify travel destination __________________________
50. Within five days prior to your illness, were you in contact with anyone who had diarrhoea (with or without vomiting)? Yes No
51. If yes, select all that apply?
52. Household
53. Health Facility
54. School
55. Neighbourhood
56. Others (specify)____________________________________________
57. Did you attend any gathering (wedding, festival, funeral etc) within five days prior to onset of your illness? Yes No
58. If yes, specify event____________________ location of event__________ Date of event_____________________________

**SECTION D: General awareness**

1. Have you heard of cholera before? Yes No
2. What do you know about cholera?
3. Cause __________________________
4. Transmission _____________________________________________
5. Symptoms (2 or 3) ________________________________________
6. Prevention______________________­­­­­_________________________
7. Treatment ________________________­­­­­­­­­­­­­­­­­­­­­­­­­­_____________________
